# Acquired Umbilico-Inguinal Fistula with Persistent Discharge due to Suture Reaction: A Case Report and Review of the Literature

**DOI:** 10.1155/2012/216345

**Published:** 2012-10-04

**Authors:** Muazez Cevik

**Affiliations:** Department of Pediatric Surgery, Faculty of Medicine, Harran University, 63100 Sanliurfa, Turkey

## Abstract

The aim of this paper is to stay a very rare umbilico-inguinal fistula (UIF) resulting from a delayed suture reaction after the use of silk suture to repair an inguinal hernia. A 3-year-old boy presented with persistent umbilical discharge. The initial diagnosis was omphalitis and he was treated with broad-spectrum antibiotics but a UIF was subsequently diagnosed. Surgery was performed to ascertain the cause of the UIF. This case demonstrates that silk suture used in inguinal hernia repair can lead to a UIF, which should be considered in the differential diagnosis of a patient presenting with persistent umbilical discharge.

## 1. Introduction

Umbilical discharge is common in infants and children and its major causes include urachus, omphalomesenteric duct remnants, granuloma, and infection of the umbilical artery. However, more rare causes exist that can be difficult to diagnose. When persistent umbilical discharge does not respond to conservative treatment, surgical exploration becomes necessary [1–3]. Inguinal hernia repair is the most common elective operation performed by paediatric surgeons, and an umbilico-inguinal fistula (UIF) is an extremely rare complication that can be caused by the use of nonabsorbable silk suture material during the procedure, which is still used at a few centres. The present study documents an unusual case of UIF and highlights the importance of selecting the correct suture material. 

## 2. Case Report

**Figure 1:**
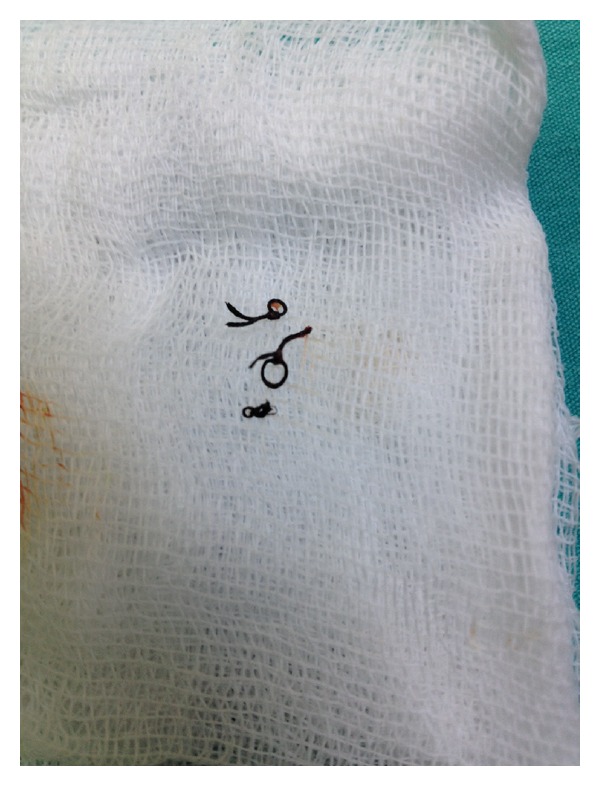
The view of foreign body as material of stitch.

A 3-year-old boy presented with umbilical discharge of 2 months' duration. The umbilicus was slightly inflamed with yellowish discharge. A scar from an inguinal hernia repair was seen on the right groin. Urinalysis and blood biochemistry were normal. Direct abdominal radiography and abdominal ultrasound were normal. During followup, swelling was noticed at the incision site and recurrent hernia was suspected. Purulent discharge was drained from the umbilicus and the swelling was treated, after which surgery was performed to lance and flush the abscess. Under general anaesthesia, an incision was made at the previous incision site. Surgical exploration showed silk suture material below the Scarpa's fascia at the level of the external inguinal ring (Figure 1). The fistula tract opened at the umbilicus over the fascia and was not connected to the peritoneum. The fistula tract was washed with copious physiologic serum until the discharge ran clear, after which a Penrose drain was placed and the incision was closed. Operative signs were consistent with fistula formation associated with delayed foreign body reaction. Appropriate antibiotic therapy was initiated and the drain was removed once the discharge resolved. The patient remained asymptomatic during the following year.

## 3. Discussion

Surgeons usually select a suture material based on their knowledge, experience, and the available materials. Suture materials have a varying risk of infection due to different physical properties and structure [4], and every suture material causes a severe inflammatory reaction in the first 7 days [5–7]. The reaction against absorbable sutures continues until the sutures are completely broken down. The major disadvantages of silk and other multifilament suture materials are that they often remain in the tissues for years as foreign bodies, allowing capillary penetration of bacteria, which can cause an acute or delayed infection by initiating an inflammatory process together with other fluids in the tissue. Consequently, they may cause abscess, granuloma, and fistula at the incision site in the early period or in other regions of the body in the late period [1, 2, 8]. However, such complications are rare in the umbilicus. In the previous study claimed the patient who had UIF the lateral umbilical ligament guided the fistula tract [9]. Because of that ligament runs alongside the inferior epigastric artery and becomes fibrotic after birth. It extends from the inguinal ring to the arcuate line, which allows the passage of the ductus deferens, testicular artery, and other components of the spermatic cord in the male, or the round ligament of the uterus in the female. Abnormal regression of fetal anatomical structures may predispose to an inguinal fistula. Another factor that may lead to formation of a fistula is weakened connective tissue resulting from an inguinal hernia.

 The present case and five cases from the literature are illustrated in Table 1. All the patients were boys who underwent surgery in the first year of life and developed an infection of their incision. However, the case described here had no postoperative complications. In the other cases, the fistula tract was removed, but in our case, the fistula tract was left in place and washed with copious saline, and a drain was placed to allow drainage during recovery. Under these conditions, the lateral umbilical ligament may fibrose. Swelling and/or infection in the inguinal region may cause opening of this structure, leading to a fistula. The ligament usually closes later in the male than the female, which explains why the majority of cases are male.

 In conclusion, when persistent umbilical discharge is observed following an inguinal hernia repair, a foreign body reaction against the suture material should be considered in the differential diagnosis.

## Figures and Tables

**Table 1:** The cases of acquired umbilical fistula after repair of inguinal hernia with the present case.

Case no.	Publication	Age	Sex	Side	Age of initial operation	Wound infection	Treatment
1	Yokomori et al. (1979) [5]	3 y	Male	Right	1 mnt	Yes	Fistulectomy
2	Yokomori et al. (1979) [5]	1 y	Male	Left	3 mnt	Yes	Fistulectomy
3	Okuyama et al. (1998) [6]	2 y	Male	Right	8 mnt	Yes	Fistulectomy
4	Daglı and Sehiraltı (1990) [9]	7 y	Male	Left	3 mnt	No	Fistulectomy
5	Daglı and Sehiraltı (1990) [9]	4 y	Male	Right	36 mnt	No	Fistulectomy
6	Present Case	3 y	Male	Right	10 mnt	No	Placed the drain in the fistula tract
